# Shift in the Microbial Ecology of a Hospital Hot Water System following the Introduction of an On-Site Monochloramine Disinfection System

**DOI:** 10.1371/journal.pone.0102679

**Published:** 2014-07-17

**Authors:** Julianne L. Baron, Amit Vikram, Scott Duda, Janet E. Stout, Kyle Bibby

**Affiliations:** 1 Department of Infectious Diseases and Microbiology, University of Pittsburgh, Graduate School of Public Health, Pittsburgh, Pennsylvania, United States of America; 2 Special Pathogens Laboratory, Pittsburgh, Pennsylvania, United States of America; 3 Department of Civil and Environmental Engineering, University of Pittsburgh, Swanson School of Engineering, Pittsburgh, Pennsylvania, United States of America; 4 Department of Computational and Systems Biology, University of Pittsburgh Medical School, Pittsburgh, Pennsylvania, United States of America; Charité-University Medicine Berlin, Germany

## Abstract

Drinking water distribution systems, including premise plumbing, contain a diverse microbiological community that may include opportunistic pathogens. On-site supplemental disinfection systems have been proposed as a control method for opportunistic pathogens in premise plumbing. The majority of on-site disinfection systems to date have been installed in hospitals due to the high concentration of opportunistic pathogen susceptible occupants. The installation of on-site supplemental disinfection systems in hospitals allows for evaluation of the impact of on-site disinfection systems on drinking water system microbial ecology prior to widespread application. This study evaluated the impact of supplemental monochloramine on the microbial ecology of a hospital’s hot water system. Samples were taken three months and immediately prior to monochloramine treatment and monthly for the first six months of treatment, and all samples were subjected to high throughput Illumina 16S rRNA region sequencing. The microbial community composition of monochloramine treated samples was dramatically different than the baseline months. There was an immediate shift towards decreased relative abundance of Betaproteobacteria, and increased relative abundance of Firmicutes, Alphaproteobacteria, Gammaproteobacteria, Cyanobacteria and Actinobacteria. Following treatment, microbial populations grouped by sampling location rather than sampling time. Over the course of treatment the relative abundance of certain genera containing opportunistic pathogens and genera containing denitrifying bacteria increased. The results demonstrate the driving influence of supplemental disinfection on premise plumbing microbial ecology and suggest the value of further investigation into the overall effects of premise plumbing disinfection strategies on microbial ecology and not solely specific target microorganisms.

## Introduction

Drinking water distribution systems, including premise plumbing, contain a diverse microbiological population [1]. Once new pipes have been added to an existing system, microbial colonization begins rapidly, with microbial communities being established in as little as one year [2]. For the purposes of this study, the ‘microbial community’ is defined as planktonic microbes within the hospital hot water system during the study period. The microbial ecology of drinking water distribution systems varies widely, depending upon system parameters such as disinfection scheme [3], hydraulic parameters [4], location in the system, age of the system [5], and pipe materials [6]. Microbes are capable of corroding pipes within distribution systems, possibly releasing harmful chemicals such as lead [7]–[9]. It is largely believed that within a drinking water distribution system, the disinfection scheme is one of the primary factors controlling the abundance and make-up of microbes [3], [6], [10]. Additionally, the effectiveness of disinfection in removing pathogens from drinking water is mediated by the microbial ecology of the drinking water system [1]. However, the impact of on-site disinfection on premise plumbing microbial ecology is not well understood, motivating the current study.

The complex microbial ecology of premise plumbing systems can serve as a reservoir for opportunistic pathogens, such as *Legionella* spp., non-tuberculous Mycobacteria, *Pseudomonas* spp., *Acinetobacter* spp., *Stenotrophomonas* spp., *Brevundimonas* spp., *Sphingomonas* spp., and *Chryseobacterium* spp. [11]–[13]. Biofilms and amoeba within the water system can protect opportunistic pathogens from disinfection [1], [14]–[16], and may even allow their regrowth and increase in pathogenicity [17]–[19]. As an example of the utility of microbial ecology-based approaches, a recent landmark microbial ecology-based study showed that biofilms in showerheads are actually enriched in opportunistic pathogens, creating the potential for an aerosol route of infection [20]. Additionally, antibiotic resistance genes have been detected in the biofilms of drinking water distribution systems [21], [22]. Each of these points highlight the necessity for a greater understanding of premise plumbing microbial ecology.

Premise plumbing systems have an approximately ten-times greater microbial load than full-scale drinking water distribution systems, due to many factors including greater water stagnation and surface area to volume ratio [23], [24]. Premise plumbing systems of hospitals are of particular concern, as hospitals may contain immunocompromised patients [25], who may not be protected by current drinking water monitoring standards [26], and who would be more susceptible to infections caused by opportunistic pathogens. To date, the majority of on-site disinfection systems have been installed in hospitals, creating a valuable testing ground to observe the impact of on-site disinfection systems on premise plumbing microbial ecology prior to more widespread application.

In addition to use in on-site systems, monochloramine as a secondary disinfectant has been advocated in the US as an effective method to reduce the production of disinfection-by-products [27], [28] and control biofilm growth within water distribution systems [29]. While monochloramine is able to penetrate biofilms better than alternative disinfectants, this may not result in a reduction in biofilm growth [8]. Additionally, chloramine treatment requires the addition of an excess of ammonia, which may cause increased growth by ammonia-oxidizing bacteria [28], such as members of the genera *Nitrospira* spp. and *Nitrosomonas* spp. [30]. Bacterial nitrification is known to increase the degradation rate of monochloramine [31], thereby reducing the expected longevity and effectiveness of chloramine. Denitrifying bacteria have previously been identified in chloraminated drinking water systems [32]; however, this topic has not been fully explored in the literature.

The effectiveness of chloramination in removing opportunistic pathogens in premise plumbing remains unclear [27]. On-site monochloramine addition has been proposed as a disinfection strategy for the control of *Legionella* [33]–[36], but long-term studies have not yet been conducted [33], [34]. Recently, a culture-based study of monochloramine on-site disinfection in a hospital’s hot water system for the purpose of *Legionella* control demonstrated a significant reduction in *L. pneumophila* and no change in nitrate or nitrite levels [37]. Observed discrepancies in system performance are potentially due to differing microbial ecologies or water chemistries of the systems tested. A more holistic view of system microbial ecology, such as presented in this study, may allow more efficient application of supplemental disinfection.

Despite the obvious importance of the microbial ecology of drinking water systems in modulating disinfectant effectiveness and as a reservoir for opportunistic pathogens, there is a notable lack of studies detailing the shift in microbial diversity and composition in response to on-site disinfection. The objective of this study was to determine the effects of on-site monochloramine disinfection on the microbial ecology of a hospital hot water system. Both the microbial ecology of hot water systems and the response of premise plumbing microbial ecology to on-site disinfection are not currently well described in the literature. This study utilizes 216 samples taken from 27 sites and pooled into five composites for two time points prior to and six time points following the addition of on-site monochloramine addition. Samples were analyzed utilizing Illumina DNA sequencing of the microbial community 16S rRNA region and results demonstrate a dynamic shift of the microbial ecology of a hospital’s hot water system in response to monochloramine addition.

## Materials and Methods

### Hospital setting

For these activities no specific permissions were required for these locations. This study took place in a 495-bed tertiary care hospital complex in Pittsburgh, PA. The building has 12 floors and receives chlorinated, municipal cold water. The hospital’s hot water system was treated with the Sanikill monochloramine injection system (Sanipur, Lombardo, Flero, Italy). Monochloramine was dosed to a target concentration between 1.5 and 3.0 ppm as Cl_2_. Details regarding monochloramine dosing and water chemistry are included in Text S1.

### Sample collection and processing

Hot water was collected from 27 sites throughout the hospital at two time points before monochloramine injection (three months and immediately prior) and monthly for the first six months of monochloramine application. Water samples were collected from a variety of locations throughout the hospital (Table 1). Samples were taken from hot water tanks, the hot water return line, faucets in the intensive care units, rehabilitation suites including both automatic and standard faucets, and other patient rooms on the upper floors. The faucets in the intensive care units are located on the third, fourth, and fifth floors. The faucets in the rehabilitation suites are located on floors six and seven and represent both electronic sensor (automatic) faucets and standard faucets. The final grouping of sites was from short-term use patient rooms located on floors eight, nine, ten, eleven, and twelve. At each site, hot water was flushed for one minute prior to sample collection into sterile HDPE bottles with enough sodium thiosulfate to neutralize 20 ppm chlorine (Microtech Scientific, Orange, CA). For hot water tank sampling, the drain valve was opened, allowed to flush for one minute, then sampled into sterile HDPE bottles as described above. Following sampling, 100 mL of sample water was filtered through a 0.2 µm, 47 mm, polycarbonate filter membrane (Whatman, Florham Park, NJ), placed into 10 mL of the original water sample, and vortexed vigorously for 10 seconds as described in methods ISO Standards 11731∶1998 and 11731∶2004 for *Legionella* isolation. Five mL of each concentrated sample was frozen at −80°C until DNA extraction.

**Table 1:** Sample pool description, abbreviati**on, and number of pooled sites.**

Sample Description	Sample Abbreviation	Number of Pooled Sites
Outlets of Hot Water Tanks and Hot Water Return Line	HWT	3
Floors 3–5 Patient Room Faucets	F3	4
Floors 6 & 7 Patient Room Automatic Faucets	F6A	7
Floors 6 & 7 Patient Room Standard Faucets and Showers	F6S	7
Floors 8–12 Patient Room Faucets	F8	6
Technical Replicates of Floors 8–12 Patient Room Faucets	F8rep	6

Hot water was collected after a one-minute flush from the following locations throughout the hospital.

### DNA extraction, PCR, and Sequencing

Frozen water samples were thawed and pooled as described in Table 1. The 27 samples were divided into five pools including the hot water tanks and hot water return line (HWT), floors 3–5 (the intensive care units, F3), floors 6 and 7 automatic faucets (the rehabilitation suites’ automatic faucets, F6A), floors 6 and 7 standard faucets (the rehabilitation suites’ standard faucets, F6S), and floors 8–12 (the short-term use patient rooms, F8). These samples were then filtered through 0.2 µm, 47 mm, Supor 200 Polyethersulfone membranes (Pall Corporation), housed in sterile Nalgene filter funnels (Thermo Scientific; Fisher). Filter membranes were subjected to DNA extraction using the RapidWater DNA Isolation Kit (MO-BIO Laboratories) as described by the manufacturer. PCR was performed in quadruplicate using 16S rRNA region primers 515F and 806R including sequencing and barcoding adapters as previously described [38]. These primers amplify an approximately 300 base pair region of the rRNA region spanning variable regions 3 and 4. The specificity of this primer set is considered to be well optimized and ‘nearly universal’ [39]; analysis of these primers against the 97% Greengenes 13.5 OTU database demonstrated a specificity of 99.9% and 98.3% for the 515f and 806r primers, respectively. Dreamtaq Mastermix (Thermo Scientific) was used and PCR product was checked on a 1% agarose gel. An independent negative control was run for each sample and primer set and all negative controls were negative for PCR amplification. PCR products were pooled and purified using the UltraClean PCR Clean-Up Kit (MO-BIO Laboratories). Each sample then underwent additional cleaning with the Agencourt AMPure XP PCR purification kit (Beckman Coulter) and quantified using the QuBit 2.0 Fluorometer (Invitrogen). Following quantification, 0.1 picomoles of each sample PCR product were pooled. The sample pool underwent two additional clean up steps with a 1.5∶1 ratio of Agencourt AMPure XP beads followed by a 1.2∶1 bead ratio (Beckman Coulter) to eliminate primer dimers. Samples were sequenced on an in-house Illumina MiSeq sequencing platform as previously described [38].

### Data analysis

Data was analyzed within the MacQIIME (http://www.wernerlab.org/software/macqiime) implementation of QIIME 1.7.0 [40]. Sequences were parsed based upon sample-specific barcodes and trimmed to a minimum quality score of 20. Operational taxonomic units (OTUs) at 97% were then picked against the Greengenes 13.5 database using UCLUST [41] for taxonomic assignment. Following assignment, 7,000 successfully assigned sequences from each sample were chosen at random to allow for even downstream analyses and even cross-sample comparison. Observed OTUs were defined as observed species whereas unassigned sequences were removed from subsequent analyses (closed reference OTU picking). Alpha-diversity evenness was calculated using the ‘equitability’ metric within QIIME. Beta diversity analyses were conducted by UNIFRAC analysis [42]. OTUs were also open-reference picked, where unassigned sequences are placed in the taxa “other” and therefore not removed. Discussion and results from this open-reference OTU picking analysis is included in Text S1. Open-reference OTU picking did not result in a shift in any fundamental conclusions with the exception of the increase in the genus *Stenotrophomonas* spp. following monochloramine addition; closed-reference OTU picking is presented for higher-quality taxonomic assignment. Morisita-Horn indices were calculated as previously described [43], [44]. Sequences are available under MG RAST accession numbers 4552832.3 to 4552878.3.

## Results

### Sequence Data

Sequencing reads were split by sample-specific barcodes, trimmed to a minimum quality score of 20, and placed into OTUs at 97% through comparison with the Greengenes 13.5 coreset. For each sample, 7,000 sequences with assigned taxonomy were selected to allow for even comparison across samples. Two types of OTU picking were done for this study: closed reference (sequences were compared to a reference set of sequences for OTU clustering, sequences not matching one of these pre-defined sequences were discarded) and open reference (sequences were compared to each other for OTU picking, sequences not mapping to the reference database were grouped as ‘other’) in Text S1.

### Alpha Diversity

**Figure 1:**
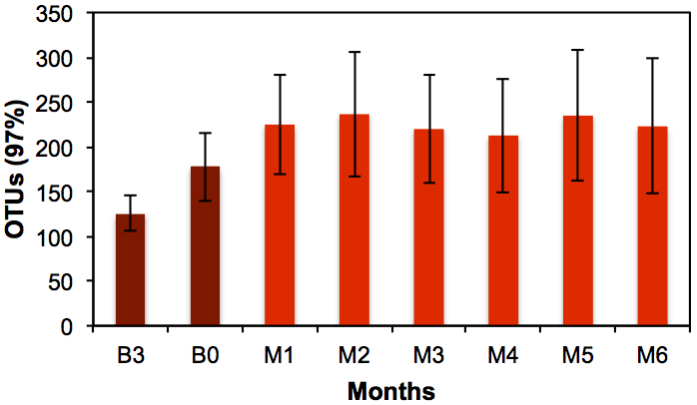
Comparison of the number of OTUs (97% similarity) for each month. Bars represent standard deviation. Each sample pool was normalized to 7,000 sequences. Samples from B3 and B0 represent those taken three months and immediately prior to monochloramine treatment, respectively. Samples from M1, M2, M3, M4, M5, and M6 were taken monthly during the first six months of treatment.

Alpha diversity (number of observed OTUs) of samples treated with monochloramine was significantly higher than samples from the baseline months (Figure 1). Prior to treatment, the average number of observed OTUs at 97% similarity was 151.2±39.7, whereas during treatment the average number of observed OTUs was 225.2±61.2 (p<0.001) (Figure 1). This shift was not associated with a statistically significant loss of sample evenness (Figure S1). The same statistical trends in alpha diversity were observed for open-reference picked OTUs (Figure S2).

### Beta Diversity

**Figure 2:**
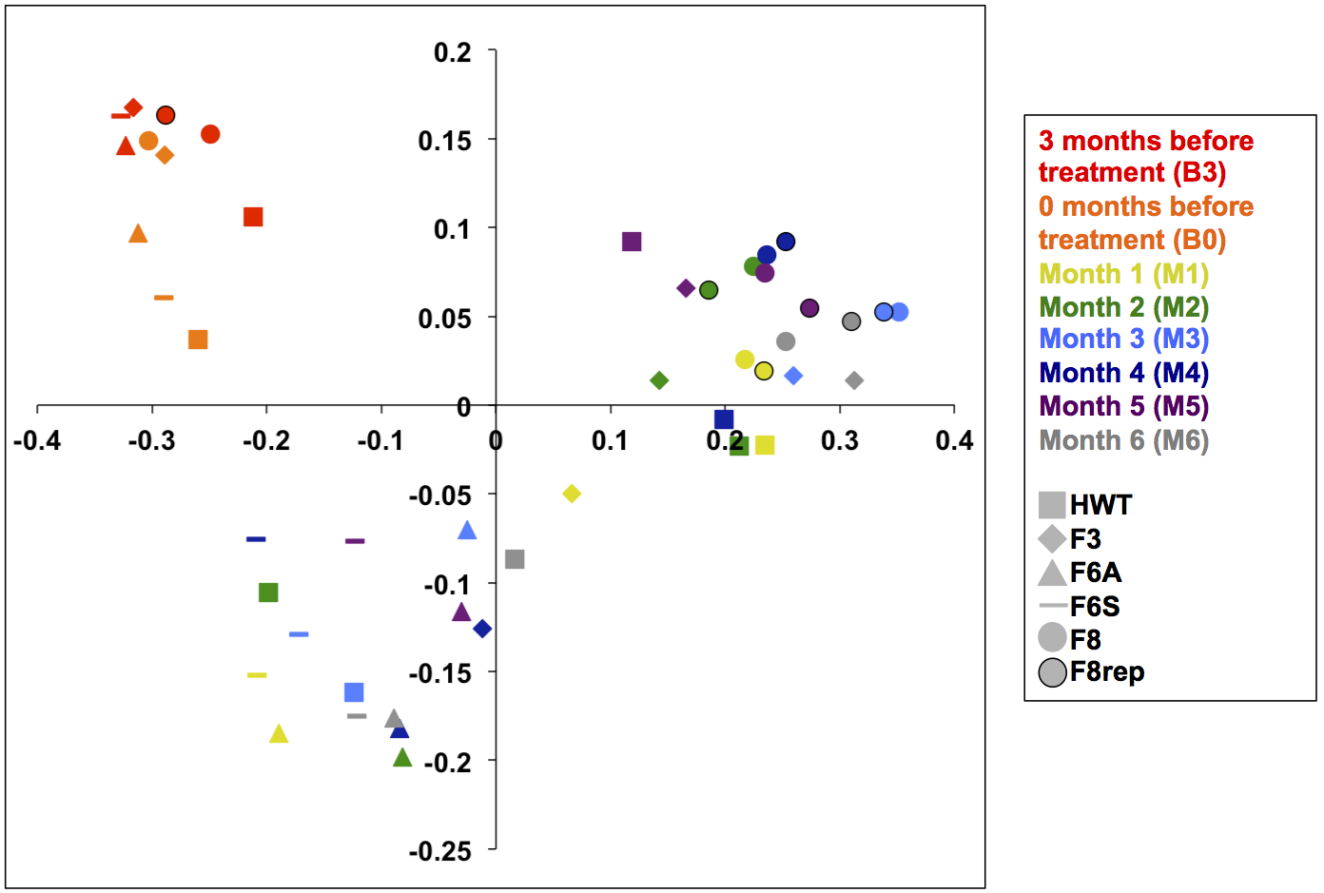
PCoA analysis of samples pools. Samples that cluster more closely together share a greater similarity in microbial community structure. Colors represent months sampled whereas shapes represent sample pool. Samples from B3 and B0 represent those taken three months and immediately prior to monochloramine treatment, respectively. Samples from M1, M2, M3, M4, M5, and M6 were taken monthly during the first six months of treatment.

Beta diversity (sample interrelatedness) was analyzed using weighted UNIFRAC [42]. The principal coordinate analysis (PCoA) plot from this analysis is shown in Figure 2. Samples from the first two months prior to treatment cluster together whereas those following disinfection tend to cluster by sample site more strongly than sample time (Figure 2). The same trend was observed for open-reference picked OTUs (Figure S3).

### Taxonomic Comparison

**Figure 3:**
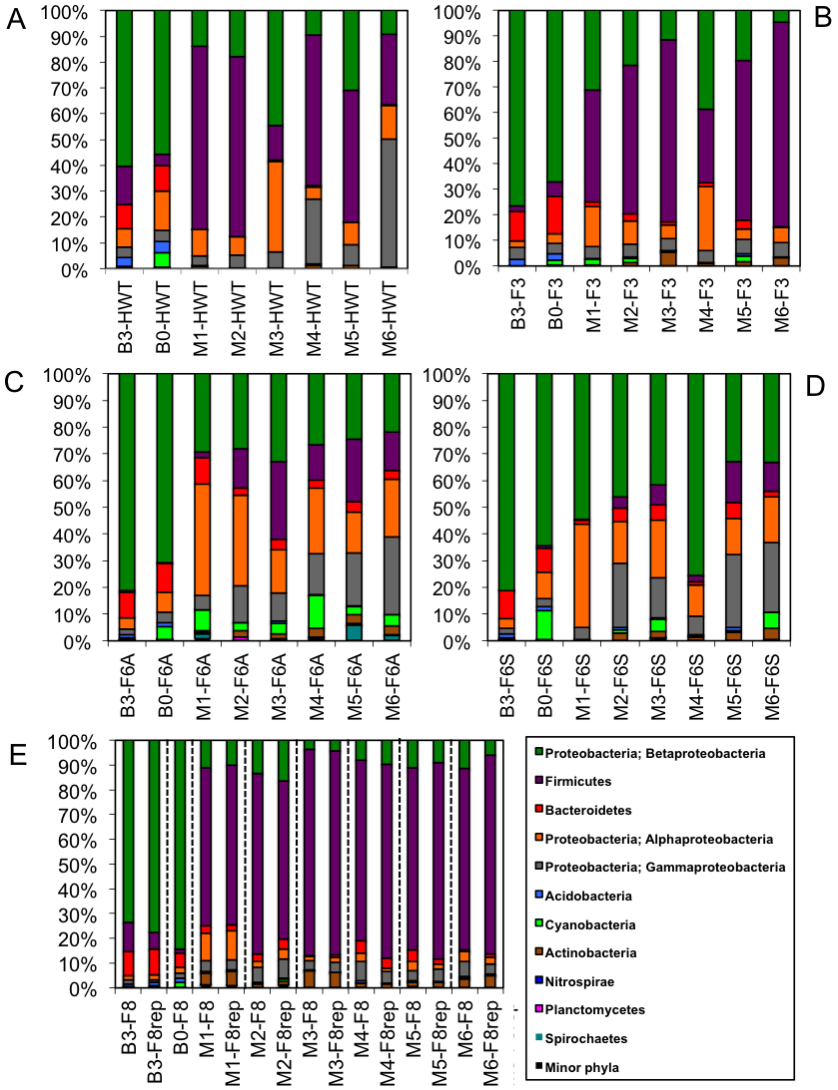
Taxonomic assignments of sequences from HWT (hot water tank samples) (Panel A), F3 (floors 3–5) (Panel B), F6A (floors 6 and 7 automatic faucets) (Panel C), F6S (floors 6 and 7 standard faucets) (Panel D), F8 (floors 8–12) and F8rep (replicate barcoded PCRs of samples from floors 8–12) (Panel E). Samples from B3 and B0 represent those taken three months and immediately prior to monochloramine treatment, respectively. Samples from M1, M2, M3, M4, M5, and M6 were taken monthly during the first six months of treatment. Black lines in Panel E separate pairs of replicates.

Figure 3 shows the phyla-level taxonomy for each of the sample pools. Phyla <1.3% relative abundance are listed as ‘minor phyla’. Prior to treatment, samples from all locations were similarly structured, predominantly comprised of Betaproteobacteria, with lesser quantities of Firmicutes, Bacteroidetes, Alphaproteobacteria, and Gammaproteobacteria (Figure 3 Panels A–E). Following initiation of treatment (M1) there was a shift away from the predominance of Betaproteobacteria and towards a greater relative abundance of Firmicutes, Alphaproteobacteria, Gammaproteobacteria, and minor fractions of Cyanobacteria and Actinobacteria (Figure 3 Panels A–E). The same taxonomy trends were observed for open-reference picked data (Figure S4 Panels A–E).

The samples from the hot water tank (HWT) from pre-treatment months (B3 and B0) were approximately 60% Betaproteobacteria with approximately 35% Firmicutes, Bacteroidetes, Alphaproteobacteria, and Gammaproteobacteria in aggregate (Figure 3 Panel A). Following treatment the relative abundance of Betaproteobacteria was reduced to approximately 20% and Firmicutes, Alphaproteobacteria, and Gammaproteobacteria subsequently increased to comprise an average of 78% of the total relative abundance (Figure 3 Panel A).

The microbial community profile of samples from the lower floors of the hospital (intensive care units, F3) was slightly different than those of the hot water tank samples but a similar trend was observed (Figure 3 Panel B). Over 65% of pre-treatment samples were Betaproteobacteria with Firmicutes, Bacteroidetes, Alphaproteobacteria, and Gammaproteobacteria accounting for a combined 20% of community relative abundance (Figure 3 Panel B). Following treatment the amount of Betaproteobacteria and Bacteroidetes decreased to an average of 23% relative abundance, while the relative abundance of Firmicutes and Alphaproteobacteria increased sharply to approximately 68% (Figure 3 Panel B).

In spite of being from the same rooms, the taxonomic composition of samples from F6A and F6S differed after treatment (Figure 3 Panels C and D). Prior to treatment both the automatic (F6A) and standard faucets (F6S) in the rehabilitation suites contained 65–80% Betaproteobacteria, with Bacteroidetes, Alphaproteobacteria, Gammaproteobacteria, and Cyanobacteria accounting for the other 20–35% of relative abundance (Figure 3 Panels C and D). However, after monochloramine application, the automatic faucets (F6A) underwent a 50% reduction in the total relative abundance of Betaproteobacteria and became enriched in Firmicutes, Alphaproteobacteria, Gammaproteobacteria, Actinobacteria, and Spirochaetes (Figure 3 Panel C). The standard faucets (F6S) lost only 26% of Betaproteobacteria, but also saw an increase in members of the Firmicutes, Alphaproteobacteria, Gammaprotobacteria, and Actinobacteria phyla from an average relative abundance of 10% before treatment to 46% after monochloramine addition (Figure 3 Panel D).

Prior to treatment, the microbial community in samples from the upper floors of the hospital (short-term use patient rooms, F8) resembled most of the other baseline samples with over 70% Betaproteobacteria and approximately 20% of Firmicutes, Bacteroidetes, Alphaproteobacteria, Gammaproteobacteria, Acidobacteria, and Cyanobacteria (Figure 3 Panel E). Following monochloramine treatment, the relative abundance of Betaproteobacteria was reduced from approximately 70% to 10% and replaced by Firmicutes, which increased from 7% of the relative abundance in the baseline months to 74% after treatment (Figure 3 Panel E). There was only a slight increase, from 2% to 9% relative abundance, in the amount of Gammaproteobacteria and Actinobacteria present (Figure 3 Panel E).

### Sample Replicates

Separately amplified and barcoded technical replicates of sample pool F8 for 7 of the 8 sample pools were also sequenced to verify technical reproducibility. There is no replicate for month B0. UNIFRAC analysis demonstrated that the replicates from each month cluster very closely (Figure 2). All of the samples from F8 in samples M1–M6 and their replicates (circles and outlined circles) clustered together in the upper-right hand quadrant (Figure 2). Morisita-Horn analyses of replicates demonstrate high levels of community similarity, ranging from 0.990 (M2) to 0.9998 (M3). These results further validate the technical reproducibility of the methodology (Figure 3 Panel E) [43], [44]. The open-reference picked UNIFRAC analysis and taxonomy also show replicates to have similar profiles to their original samples (Figure S3 and S4 Panel E). Morisita-Horn analyses of these samples showed similarly high levels of community similarity ranging from 0.991 (M2) to 0.9992 (M1).

### Genera Containing Opportunistic Pathogens

**Figure 4:**
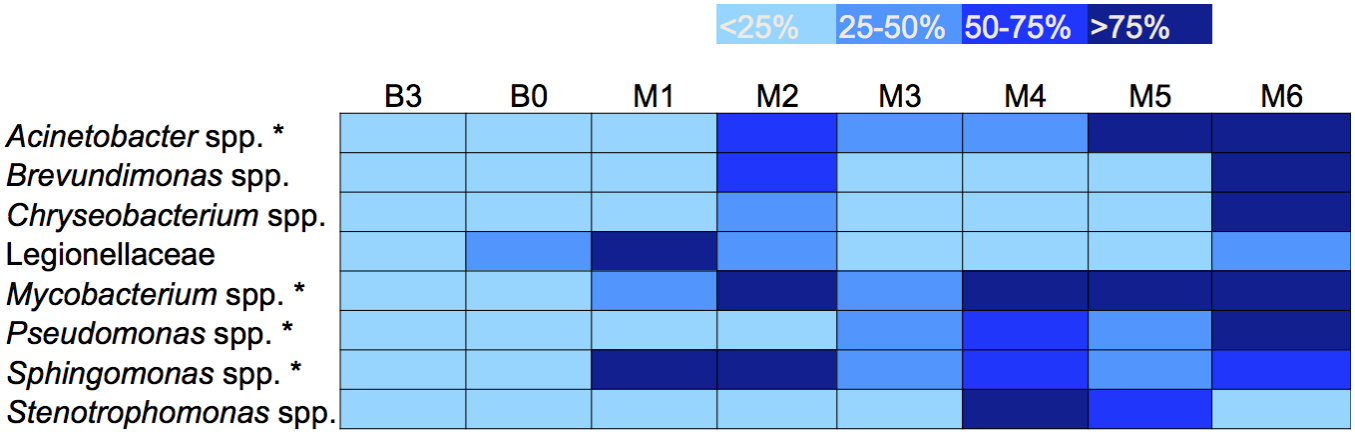
Relative abundance of different genera of opportunistic waterborne pathogens. Samples color coded into four groupings calculated by 25% of the maximum relative abundance for each organism. Months with the least relative abundance are lightest in color, whereas months with the highest relative abundance are darkest. *denotes a statistically significant increase in the relative abundance of this organism following treatment.

Sequence data was further analyzed to observe the change in genera containing opportunistic pathogens of interest during treatment. Genera analyzed were: *Legionella* spp., *Pseudomonas* spp., *Acinetobacter* spp., and *Stenotrophomonas* spp. (Gammaproteobacteria group); *Brevundimonas* spp. and *Sphingomonas* spp. (Alphaproteobacteria group); *Chryseobacterium* spp. (Bacteroidetes group); and *Mycobacterium* spp. (Actinobacteria group). These genera are of special interest as some to all of the species contained within them are pathogens; however, the nature of short-read 16S rRNA region sequence analysis is such that species-level pathogens cannot be definitively identified. Trends demonstrated by this analysis could be used to direct future analyses targeting opportunistically pathogenic organisms more specifically. Analysis of the relative abundance of each of these organism groups over time shows a statistically significant increase in relative abundance for *Acinetobacter* (p = 0.0054), *Mycobacterium* (p = 0.0017), *Pseudomonas* (p = 0.031) and *Sphingomonas* (p = 0.034) as treatment progressed (Figure 4). *Brevundimonas*, *Chryseobacterium*, Legionellaceae, and *Stenotrophomonas* did not demonstrate a statistically significant increase in relative abundance following treatment (Figure 4). The open-reference picked data demonstrated an increase in the same opportunistic pathogen containing genera as the closed-reference picked data, *Acinetobacter* (p = 0.004), *Mycobacterium* (p = 0.002), *Pseudomonas* (p = 0.015), and *Sphingomonas* (p = 0.025), but also showed a significant increase in the genera *Stenotrophomonas* (p = 0.03) (Figure S5).

### Nitrification and Denitrification

**Figure 5:**
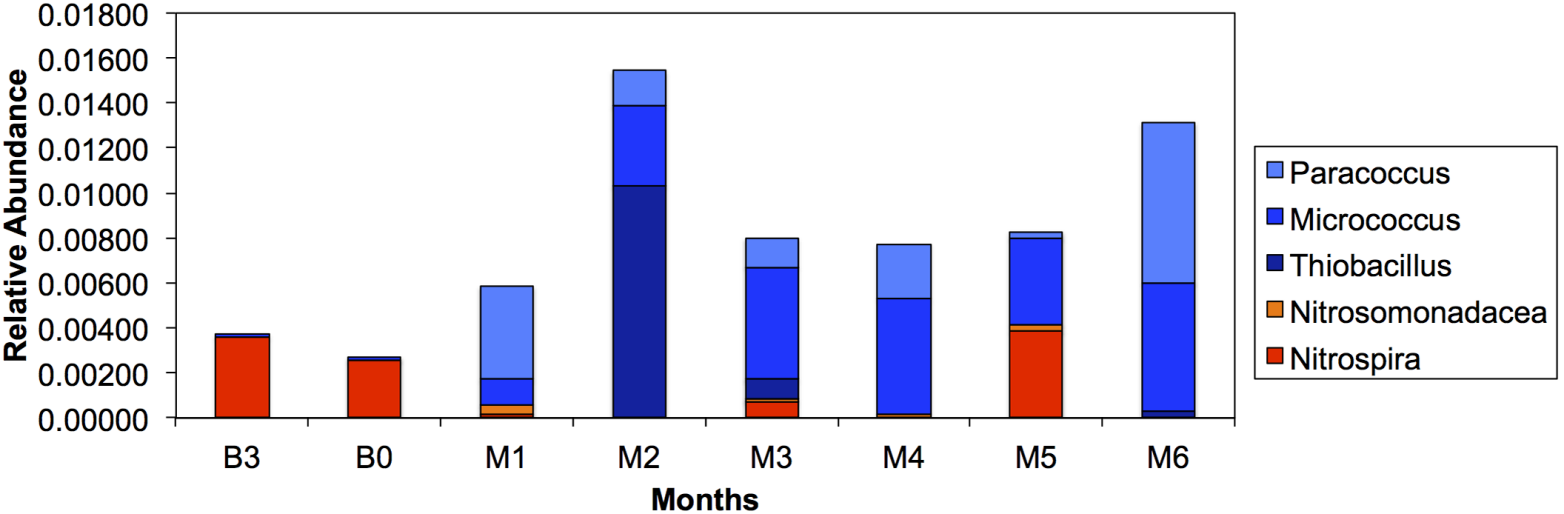
Relative abundance of genera containing nitrifying (*Nitrospira* and Nitrosomonadacea) and denitrifying bacteria (*Thiobacillus*, *Micrococcus*, and *Paracoccus*). No other genera associated with nitrification (*Nitrosococcus*, *Nitrobacter*, *Nitrospina*, or *Nitrococcus*,) or denitrification (*Rhizobiales* and *Rhodanobacter*) were found in any of our samples. The x-axis represents sampling months with months B3 and B0 being before monochloramine treatment and months M1–M6 representing the first six months of treatment. The y-axis represents the relative abundance.

Additionally, we investigated the shift in relative abundance of representative genera associated with nitrification and denitrification (Figure 5). There was no statistically significant difference in the relative abundance of the potential nitrifiers *Nitrospira* and Nitrosomonadaceae, before (mean = 0.0015±0.0018) and after treatment (mean = 0.0005±0.0011) (p = 0.175). Other nitrifier-containing genera such as *Nitrosococcus*, *Nitrobacter*, *Nitrospina*, or *Nitrococcus*, were not identified in any samples. The total relative abundance of genera containing denitrifiers (*Thiobacillus, Micrococcus*, and *Paracoccus*) underwent a statistically significant increase before (mean = 0.00005±0.000074) and after treatment with monochloramine (mean = 0.0029±0.0029) (p = 0.026). The denitrifier-containing genera *Rhizobiales* and *Rhodanobacter* were not identified in any samples. The same trends were observed in open-reference picked data (Figure S6).

## Discussion

Our study objective was to examine the shift in the microbial ecology of a hospital hot water system associated with the introduction of on-site monochloramine addition. To evaluate the shift in microbial community structure we sampled 27 sites in a hospital and pooled samples into 5 groups for 8 sample time points. Sites were pooled based on their location and use in the hospital and faucet type (automatic versus standard). This study took place during the first U.S. trial of the Sanikill on-site monochloramine generation system (Sanipur, Brescia, Italy) [45]–[47]. These samples were subjected to DNA extraction, 16S rRNA region barcoded PCR, and Illumina sequencing to analyze the response of the microbial ecology to the addition of monochloramine.

The microbial population shift in response to monochloramine addition was immediate. The number of OTUs observed (alpha diversity) significantly increased following monochloramine treatment (Figure 1). It is possible that the overall loss of dominance of initially abundant microbial groups (e.g. Betaproteobacteria) allowed for a greater number of other bacterial species to grow, or for selected individuals to die off, thereby increasing the alpha diversity. Samples from different sites taken before monochloramine treatment were comprised of similar microbial populations and samples taken after treatment were distinct from samples taken in the baseline months (Figures 2 and 3, Figures S3 and S4). Interestingly, it appears that following monochloramine treatment the location of sampling matters more in sample similarity (beta diversity) than does the month they were taken (Figure 2, Figure S3). Microbial communities from the lower floors’ intensive care units (F3) and the upper floors’ short term patient rooms (F8) were more similar than to the floors 6 and 7’s rehabilitation suites (F6A and F6S) automatic and standard faucet samples. These sites were located in single patient rooms in rehabilitation units and may experience as much use as some locations on the lower and upper floors, which include the trauma burn unit, the intensive care unit (ICU), the neonatal ICU, and the cardiovascular ICU. The HWT samples from earlier months of treatment closely resembled floors 6 and 7 (F6A and F6S) whereas the HWT microbial ecology from the later months was more related to the lower (F3) and upper floors (F8).

We investigated the possible differences in microbial ecology between automatic and standard faucets as it has been previously demonstrated that opportunistic pathogens, including *Legionella* [48] and *Pseudomonas aeruginosa* [49], are detected more frequently and in greater concentrations in automatic faucets. It has been suggested that the reason for the differences between automatic and standard faucets could be due to water flow, temperature, and structural issues. Automatic faucets may have diluted monochloramine concentrations due to low flow and poor flushing [48], [49] and automatic faucets also contain mixing valves, which are made of materials such as rubber, polyvinylchloride, and plastic, which more easily support the growth of biofilms [48], [49]. Potentially due to these biofilms, the increased colonization can persist even following disinfection with chlorine dioxide [48]. We observed a differential reduction in the relative abundance of Betaproteobacteria in standard and automatic faucets following treatment. The automatic faucets lost 50% of their relative abundance of Betaproteobacteria whereas the standard faucets only saw an average 26% reduction.

There was an overall shift towards less relative abundance of Betaproteobacteria, and more relative abundance of Firmicutes, Alphaproteobacteria, Gammaproteobacteria, Cyanobacteria and Actinobacteria after monochloramine treatment. A previous microbial ecology study of a simulated drinking water distribution system treated with monochloramine demonstrated a different trend, with an increase in specific genera within the Actinobacteria, Betaproteobacteria, and Gammaproteobacteria phyla [3]. The dissimilarity of these studies may be due to the fact that the latter occurred in a cold water system whereas our study was in a hot water supply.

Several waterborne pathogen-containing genera were examined for changes in relative abundance due to monochloramine treatment. The relative abundance of a few of the waterborne pathogen-containing genera examined, including *Acinetobacter*, *Mycobacterium*, *Pseudomonas*, and *Sphingomonas*, showed an increase after monochloramine treatment. Other studies have described an increase in some of these organisms including *Legionella*, *Mycobacterium*, and *Pseudomonas* in chloraminated water [3], [6] as well as biofilms treated with monochloramine [50]. Feazel et al. previously demonstrated that *Mycobacterium* spp. can be enriched in showerhead biofilms compared to the source water [20]. An increased relative abundance of *Mycobacterium* spp. due to monochloramine treatment is of concern, specifically if this increase in relative abundance is due to the presence of more viable mycobacterial cells. These microorganisms may pose a specific threat of aerosol exposure to immunocompromised patients who reside in buildings with an increased abundance of these organisms in hot water [20]. Interestingly, a recent study demonstrated that while the concentration of live bacteria is reduced after monochloramine treatment, only the viable microbial community structure is altered and genera containing opportunistic pathogens persist [51]. While we did not directly quantify microorganisms in the samples collected or verify that microorganisms detected were viable, our parallel culture-based study observed a statistically significant reduction in culturable total bacteria and *Legionella* species following monochloramine treatment (Table S1) [45]–[47], [52].

Previous studies have found an increase in nitrification in chloraminated systems, which effectively decreased monochloramine concentration [6], [31]. This chemical decay led to higher levels of *Legionella*, *Mycobacterium* spp., and *P. aeruginosa* at earlier water ages than in chlorinated simulated distribution systems [6]. A change in potentially nitrifying bacteria following monochloramine addition was not observed in the culture-based portion of this study [45]–[47], consistent with our molecular observations. Concentrations of nitrate and nitrite remained fairly stable throughout the study months, with the exception of a spike in nitrate levels in M6 (Table S1) [52]. We observed a statistically significant increase in the relative abundance of genera associated with denitrification in monochloramine treated samples. A previous study found a high absolute abundance, up to 200,000 cfu/mL, of potentially denitrifying bacteria in a chloraminated system even after regular flushing [32]. The highest relative abundance of bacterial genera associated with denitrification occurred during M6 when there was a spike in nitrate concentrations (Table S1) [52]. However, in months 1 and 2 there was also a large relative abundance of these bacteria present with fairly low nitrate concentrations, suggesting that some other factor might be important in their relative abundance. We do not believe that these trends were due to seasonality in our study as microbiological data were largely consistent across the study period. However, the possibility for seasonal effects cannot be excluded.

A notable increase in the relative abundance of the genus *Alicyclobacillus* spp. (Firmicutes phylum) was observed following monochloramine treatment, from an average of 4.1±4.5% of the microbial population prior to treatment to an average of 40.9±27.1% following treatment (p<0.001). This genera is comprised primarily of spore-formers that are of concern in food spoilage [53], and has previously been detected in drinking water [54]. The high relative abundance of *Alicyclobacillus* spp. suggests a potentially dominant role in chloraminated hot water system microbial ecology worthy of future investigation.

The incidence of reported Legionnaires’ disease cases increased threefold from 2000 to 2009 [55]. This fact, coupled with an increasingly elderly and immunocompromised population [55], has lead to an increased concern about *Leginonella* and other opportunistic waterborne pathogens. Additionally, the American Society of Heating, Refrigerating, and Air-Conditioning Engineers (ASHRAE) has recently proposed Standard 188P for the prevention of legionellosis associated with premise plumbing systems [56]. This standard serves to reduce the risk of *Legionella* infections through a risk management approach [56]. For these reasons, on-site disinfection has become progressively important to protect patients in hospitals and long-term care facilities from waterborne opportunistic pathogens. An increased understanding of the influence of on-site disinfection on premise plumbing microbial ecology is necessary to maximize effectiveness and to limit undesired side effects.

This study demonstrates that there exists the potential for unwanted consequences of supplemental disinfectant addition for the removal of *Legionella* such as the potential enrichment of other waterborne pathogens, including *Acinetobacter*, *Mycobacterium*, *Pseudomonas*, and *Sphingomonas*. Understanding the impact of supplemental disinfection on water system microbial ecology, through a holistic approach, is necessary to maximize disinfectant effectiveness and to ensure that supplemental disinfectant does not select for alternative opportunistic pathogens. A recent review emphasizes not only the role of disinfectants but also other system factors that may impact microbial ecology such as temperature, pipe material, organic carbon, presence of automatic faucets, and point-of-use filtration [24]. The authors suggest a probiotic approach to opportunistic pathogen control which would either add microbes that can outcompete these pathogens, remove key species, or using engineering controls to favor benign organisms that are antagonistic to opportunistic pathogens [24]. This systematic, probiotic, approach to premise plumbing opportunistic pathogen management is an inventive concept for dealing with the diverse microbial ecology of these systems, but requires a greater understanding of the drivers of premise plumbing microbial ecology, such as provided by this study.

In conclusion, we observed a shift in the microbial ecology of a hospital’s hot water system treated with on-site chloramination. This shift occurred immediately following monochloramine treatment. Prior to treatment, the bacterial ecology of all samples was dominated by Betaproteobacteria; following treatment, members of Firmicutes and Alphaproteobacteria dominated. Differences in community composition were seen in different locations within the hospital as well as between automatic and standard faucets. This suggests that water from different locations and outlet types should be sampled to get a more thorough picture of the microbiota of a system. There was an increase in the relative abundance of several genera containing opportunistic waterborne pathogens following the onset of monochloramine treatment, including *Acinetobacter, Mycobacterium*, *Pseudomonas*, and *Sphingomonas* and genera associated with denitrification. The benefits and risks of each supplemental disinfection strategy should be evaluated before implementation in any building, especially in hospitals, long term care facilities, and other buildings housing immunocompromised patients. This work demonstrates the effects of a supplemental monochloramine disinfection system on the microbial ecology of premise plumbing biofilms. Given the importance of premise plumbing microbial ecology on opportunistic pathogen presence and persistence, understanding the driving influence of supplemental disinfectants on microbial ecology is a crucial component of any effort to rid premise plumbing systems of opportunistic pathogens. As additional facilities turn to on-site water disinfection strategies, more long-term studies on the effects of disinfectants on microbial ecology in premise plumbing are needed as well as those evaluating a probiotic approach to opportunistic pathogen eradication.

## Supporting Information

Figure S1 **Sample evenness for closed-reference OTU picking.** No statistically significant different was observed for samples taken prior to or following monochloramine addition.
(TIF)

Figure S2 **Alpha diversity for open-reference OTU picking.** A statistically significant difference was observed for samples taken prior to or following monochloramine addition (p = 0.046).
(TIF)

Figure S3 **Beta diversity for open-reference OTU picking.** Samples from before monochloramine treatment clustered together whereas following treatment samples clustered by location more so than month of treatment.
(TIF)

Figure S4 **Taxonomic assignment of sequences from HWT (hot water tank samples) (Panel A), F3 (floors 3–5) (Panel B), F6A (floors 6 and 7 automatic faucets) (Panel C), F6S (floors 6 and 7 standard faucets) (Panel D), F8 (floors 8–12) and F8rep (replicate barcoded PCRs of samples from floors 8–12) (Panel E) for open-reference OTU picking.**
(TIF)

Figure S5 **Relative abundance of waterborne pathogen containing genera for open-reference OTU picking.** A statistically significant increase in *Acinetobacter* spp., *Mycobacterium* spp., *Pseudomonas* spp., *Sphingomonas* spp., and *Stenotrophomonas* spp. was observed following treatment.
(TIF)

Figure S6 **Relative abundance genera containing nitrifying (** ***Nitrospira*** **and Nitrosomonadacea) and denitrifying bacteria (** ***Thiobacillus*** **,** ***Micrococcus*** **, and** ***Paracoccus*** **) for open-reference OTU picking.** No other genera containing nitrifying bacteria (*Nitrosococcus*, *Nitrobacter*, *Nitrospina*, or *Nitrococcus*,) or denitrifying bacteria (*Rhizobiales* and *Rhodanobacter*) were found in our samples.
(TIF)

Table S1 **Physicochemical data obtained during the study.**
(DOCX)

Text S1 **Supplementary Information.** Water chemistry and monochloramine dosing methods, description of minor phyla observed, and open-reference OTU picking results.
(DOCX)
