# Supplementary figures and images for: Shift in the Microbial Ecology of a Hospital Hot Water System following the Introduction of an On-Site Monochloramine Disinfection System
Source: PLoS One. 2014 Jul 17;9(7):e102679. doi: 10.1371/journal.pone.0102679 (PMC4102543; doi:10.1371/journal.pone.0102679)

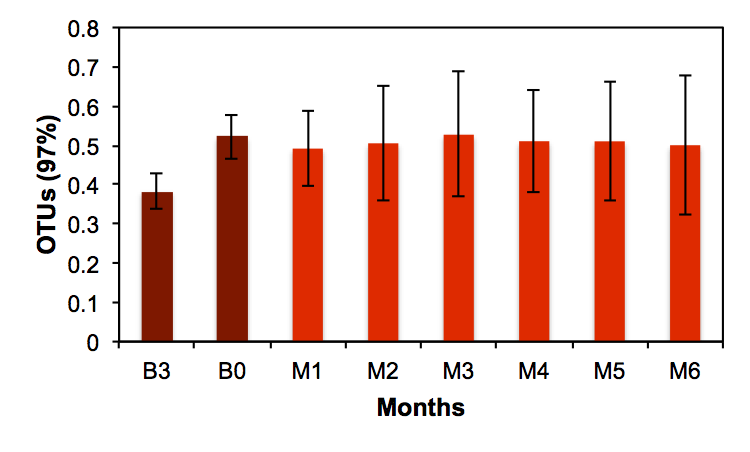

Supplement: Figure S1 — Sample evenness for closed-reference OTU picking. No statistically significant different was observed for samples taken prior to or following monochloramine addition. (TIF) [file pone.0102679.s001.tif]

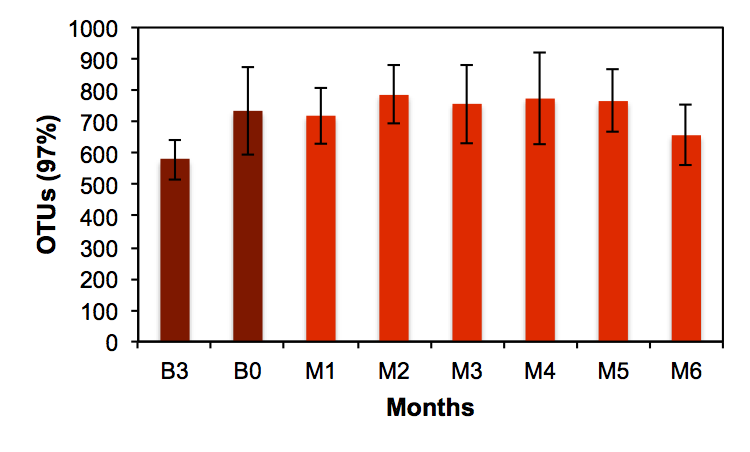

Supplement: Figure S2 — Alpha diversity for open-reference OTU picking. A statistically significant difference was observed for samples taken prior to or following monochloramine addition (p = 0.046). (TIF) [file pone.0102679.s002.tif]

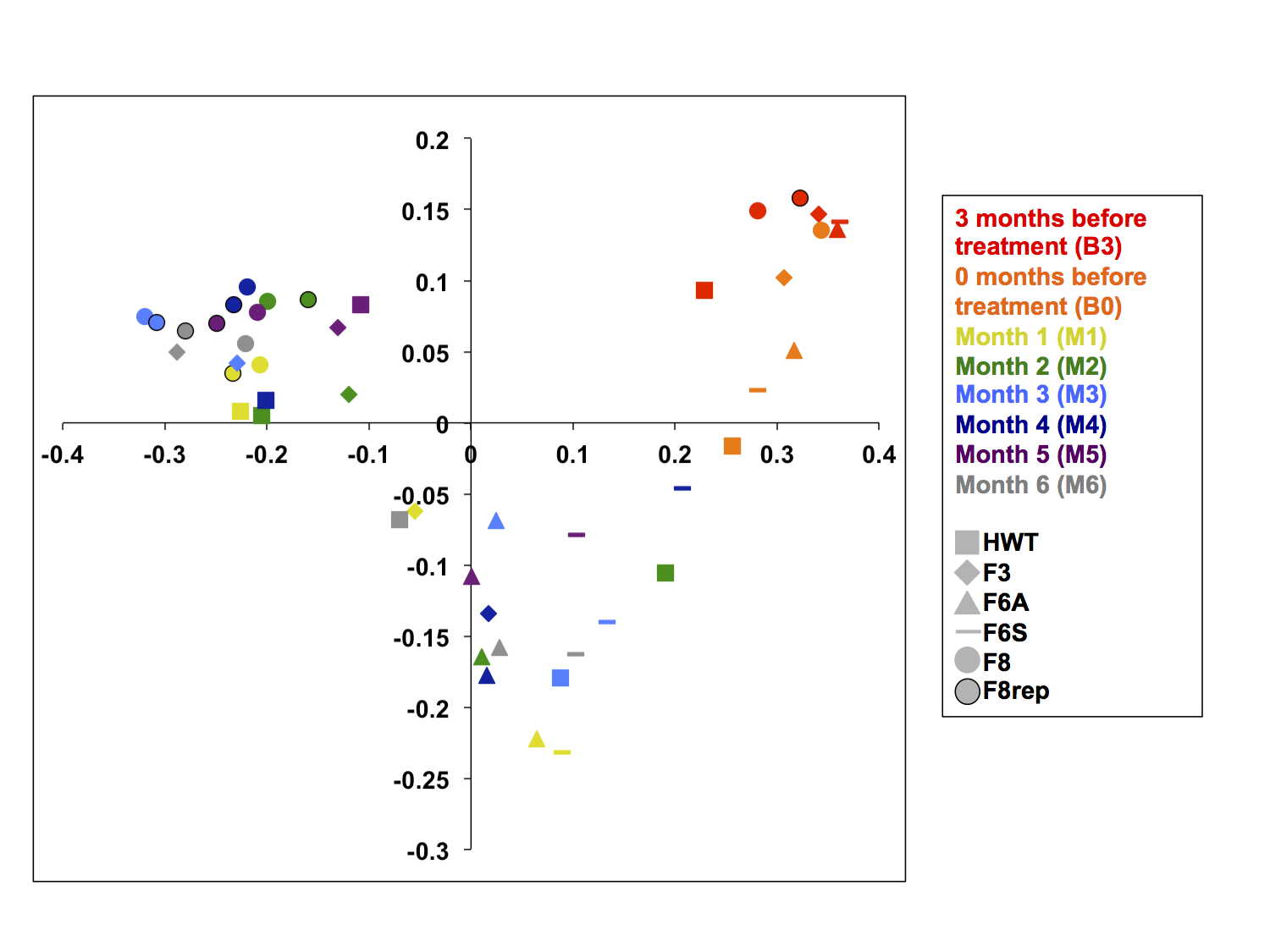

Supplement: Figure S3 — Beta diversity for open-reference OTU picking. Samples from before monochloramine treatment clustered together whereas following treatment samples clustered by location more so than month of treatment. (TIF) [file pone.0102679.s003.tif]

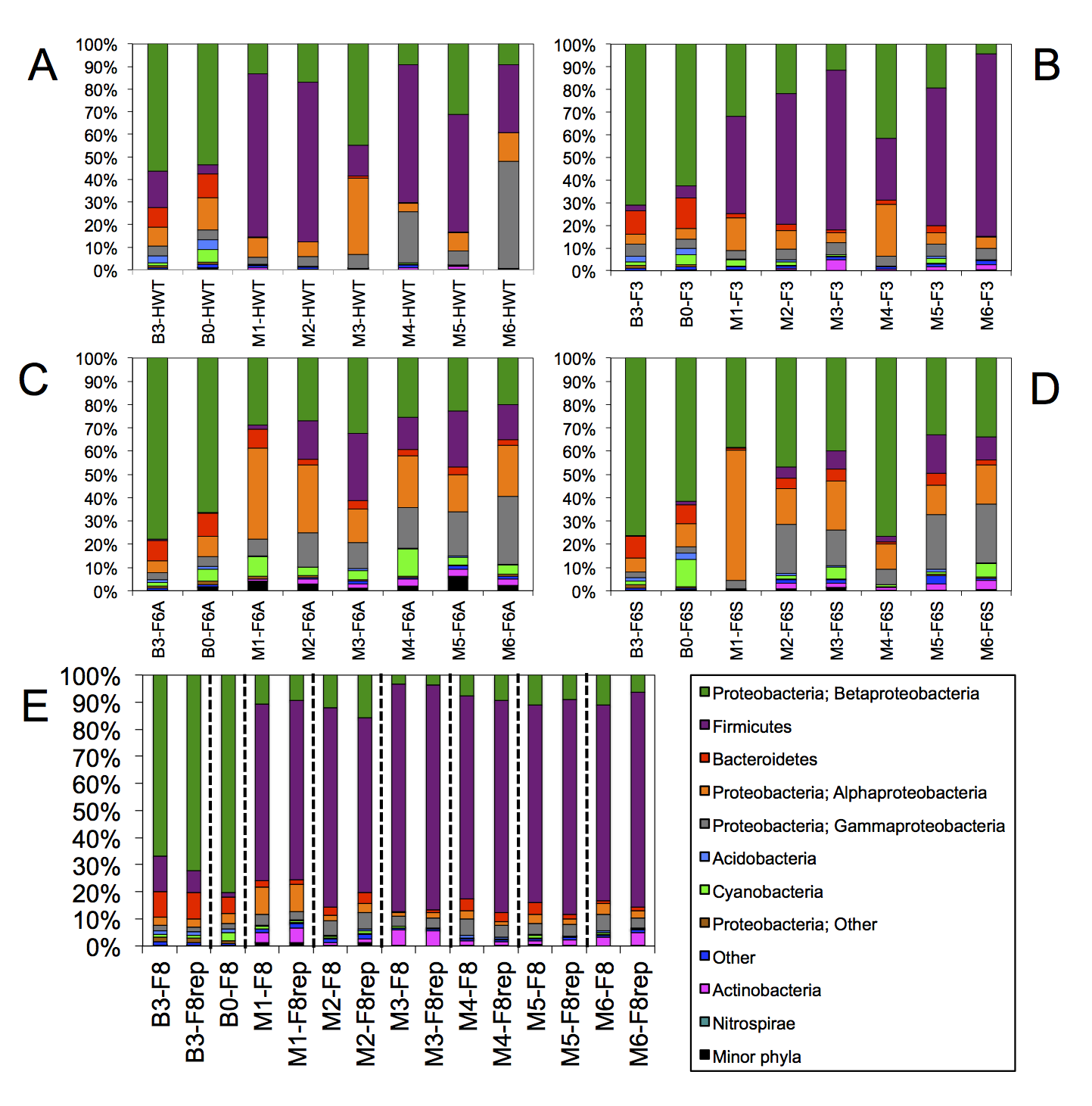

Supplement: Figure S4 — Taxonomic assignment of sequences from HWT (hot water tank samples) (Panel A), F3 (floors 3–5) (Panel B), F6A (floors 6 and 7 automatic faucets) (Panel C), F6S (floors 6 and 7 standard faucets) (Panel D), F8 (floors 8–12) and F8rep (replicate barcoded PCRs of samples from floors 8–12) (Panel E) for open-reference OTU picking. (TIF) [file pone.0102679.s004.tif]

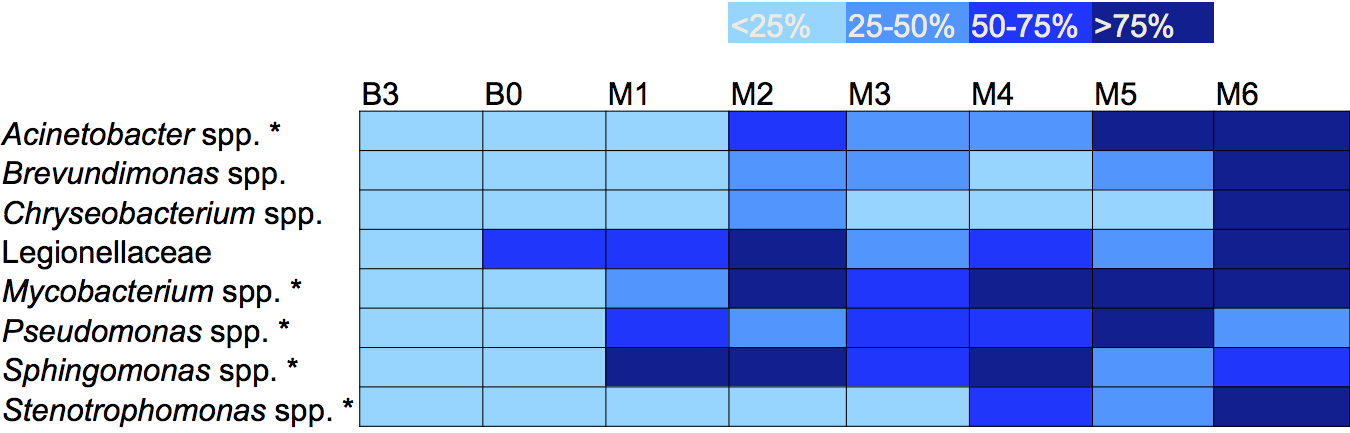

Supplement: Figure S5 — Relative abundance of waterborne pathogen containing genera for open-reference OTU picking. A statistically significant increase in Acinetobacter spp., Mycobacterium spp., Pseudomonas spp., Sphingomonas spp., and Stenotrophomonas spp. was observed following treatment. (TIF) [file pone.0102679.s005.tif]

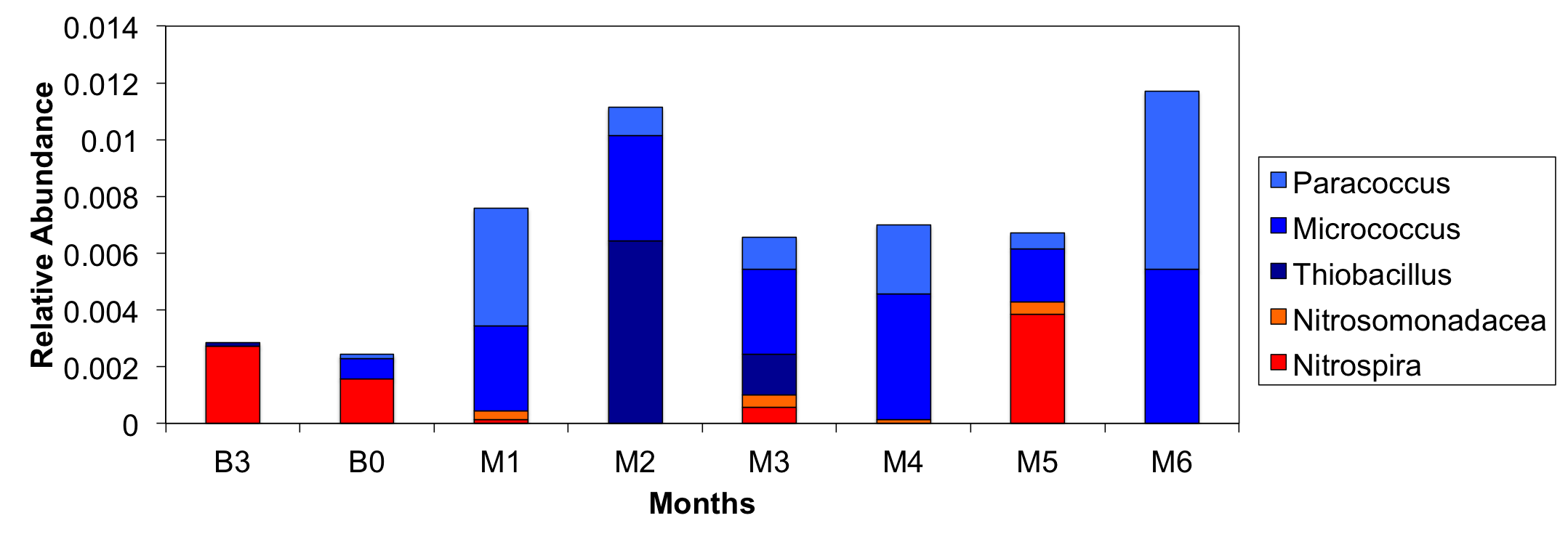

Supplement: Figure S6 — Relative abundance genera containing nitrifying ( Nitrospira and Nitrosomonadacea) and denitrifying bacteria ( Thiobacillus , Micrococcus , and Paracoccus ) for open-reference OTU picking. No other genera containing nitrifying bacteria (Nitrosococcus, Nitrobacter, Nitrospina, or Nitrococcus,) or denitrifying bacteria (Rhizobiales and Rhodanobacter) were found in our samples. (TIF) [file pone.0102679.s006.tif]
