# Supplementary material for: Shift in the Microbial Ecology of a Hospital Hot Water System following the Introduction of an On-Site Monochloramine Disinfection System
Source: PLoS One. 2014 Jul 17;9(7):e102679. doi: 10.1371/journal.pone.0102679 (PMC4102543; doi:10.1371/journal.pone.0102679)
Supplement: Table S1 — Physicochemical data obtained during the study. (DOCX) [file pone.0102679.s007.docx]

**Table S1. Physicochemical data obtained during the study [1]**

| **Parameter** | **B^a^** | **M1** | **M2** | **M3** | **M4** | **M5** | **M6** |
| --- | --- | --- | --- | --- | --- | --- | --- |
| ***Legionella* Distal Site % Positivity** | 53% | 7% | 4% | 7% | 4% | 4% | 7% |
| **Avg. HPC (log[CFU/mL])** | 4.15 | 3.87 | 2.64 | 3.01 | 3.76 | 1.55 | 2.68 |
| **pH** | 8.3 | 8.6 | 8.1 | 8.1 | 8 | 7.9 | 8.1 |
| **Monochloramine (ppm as Cl_2_)** | 0 | 3.14 | 0.76 | 1.6 | 2.58 | 2.8 | 2.57 |
| **Total Chlorine (ppm)** | 0.02 | 2.45 | 0.65 | 1.22 | 2.25 | 2.31 | 2.5 |
| **Free Chlorine (ppm)** | 0.03 | 0.14 | 0.09 | 0.15 | 0.22 | 0.17 | 0.13 |
| **Total Ammonia (ppm)** | 0.01 | 0.31 | 0.14 | 0.46 | 0.39 | 0.46 | 0.85 |
| **Nitrate (ppm)** | 0.5 | 1.1 | 0.8 | 1.4 | 1.3 | 1.5 | 6.8 |
| **Nitrite (ppm)** | 0.002 | 0.005 | 0.002 | 0.006 | 0.005 | 0.005 | 0.006 |
| **Copper (ppm)** | 0.14 | 0.15 | 0.38 | 0.36 | 0.74 | 0.76 | 0.46 |
| **Silver (ppm)** | 0.012 | 0.03 | 0.026 | 0.02 | 0.035 | 0.097 | 0.052 |
| **Lead (ppm)** | <0.0025 | <0.0025 | <0.0025 | <0.0025 | <0.0025 | <0.0025 | 0.0035 |

^a^ Baseline sampling was taken once, immediately prior to the initiation of the monochloramine generation system

**Supplementary Reference:**

1. Duda S, Kandiah S, Stout JE, Baron JL, Yassin MH, et al. (2014) Evaluation of a new monochloramine generation system for controlling *Legionella* in building hot water systems. Submitted for publication.
