# Supplementary material for: Shift in the Microbial Ecology of a Hospital Hot Water System following the Introduction of an On-Site Monochloramine Disinfection System
Source: PLoS One. 2014 Jul 17;9(7):e102679. doi: 10.1371/journal.pone.0102679 (PMC4102543; doi:10.1371/journal.pone.0102679)
Supplement: Text S1 — Supplementary Information. Water chemistry and monochloramine dosing methods, description of minor phyla observed, and open-reference OTU picking results. (DOCX) [file pone.0102679.s008.docx]

**Supporting Information: Shift in Microbial Ecology of a Hospital Hot Water System Following the Introduction of an On-Site Monochloramine Disinfection System**

by

Julianne L. Baron, Amit Vikram, Scott Duda, Janet E. Stout, and Kyle J. Bibby

**Water Chemistry and Monochloramine Dosing**

Monitoring of physicochemical parameters included pH, monochloramine, total chlorine, free chlorine, total ammonia, nitrate, nitrite, copper, silver, and lead (see Table S1). A Hach DR/890 was used for all measurements except copper, silver, and lead which were sent to a reference laboratory (Analytics Corporation, Ashland, VA) [1]. Two precursor reagents (Enoxin (stabilized sodium hypochlorite) and Zebion (buffered ammonia salt solution)) were added to a pre-dilution loop supplied by the hot water return [1]. The precursors were dosed into this loop, and treated water was then injected into the circulating hot water [1]. Samples for physicochemical analysis were taken from both the hot water return and the first post-monochloramine injection outlet. Presented values are the average of measurements from the chemical concentrations put into circulation in the hot water system (first post-injection outlet) and those remaining upon return of the hot water after passage through the building (hot water return line).

Following initiation of monochloramine treatment, both *Legionella* distal site positivity and average HPC decreased significantly (p < 0.05) (Table S1) [1]. Concentrations of nitrate, nitrite, copper, and lead did not exceed their EPA maximum contaminant levels (Table S1) [1]. Total chlorine, free chlorine, and total ammonia concentrations increased upon initiation of monochloramine injection and mirrored the variability of monochloramine levels (Table S1) [1].

**Minor Phyla**

Samples included in the ‘minor phyla’ group of the closed-reference picked OTUs include the following phyla: Armatimonadetes, Chlorobi, Chloroflexi, Crenarchaeota, Deltaproteobacteria, FBP, Fusobacteria, Gemmatimonadetes, SBR1093, TM6, TM7, Verrucomicrobia, WPS-2, TA18, and Thermi. In the open-reference picked OTUs the ‘minor phyla’ include Armatimonadetes, Chlamydiae, Chlorobi, Chloroflexi, Crenarchaeota, Deltaproteobacteria, Fusobacteria, Gemmatimonadetes, OD1, Other, Planctomycetes, SBR1093, Spirochaetes, Thermi, TM6, Verrucomicrobia, WPS-2, and WYO.

**Open reference data/figures (SI)**

It is currently unclear which approach is most appropriate by which to pick operational taxonomic units (OTU). Closed-reference OTU picking assigns sequences based on comparison with a reference database. This approach is recognized to provide the most robust taxonomic assignments but excludes sequences not matched to the database. Open-reference OTU picking first assigns sequences based on comparison with a reference database and then picks OTUs de novo from unassigned sequences. This approach is more inclusive of diversity but perceived to be less accurate taxonomic assignment. Closed-reference OTU picking results are presented in the manuscript due to advantages in taxonomic assignment; however, results from open-reference OTU picking are included here to demonstrate that conclusions are robust to OTU picking approach.

For open-reference OTU picking:

- The alpha diversity of treated samples was statistically significantly higher than baseline (Figure S2). Prior to treatment average OTUs at 97% was 656.2 ± 131.1, during treatment average number of observed OTUs was 743 ± 110.3 (p = 0.046) (Figure S2). Same conclusions as closed-reference picking.
- Samples from the first two months are clustered together, however less tightly than in closed reference analysis (Figure S3). Samples from F6A and F6S as well as a few HWTs cluster together (Figure S3). Samples from F3, F8, F8rep and half of the HWTs cluster (Figure S3). Same conclusions as closed-reference picking.
- Overall taxonomy data was the same as closed reference data (Figure S4 Panels A-E). Data from each pool shows the same pattern as closed reference data (Figure S4 Panels A-E). Same conclusions as closed-reference picking.
- Replicate PCRs cluster together in PCA analysis (Figure S3). Also taxonomy is equivalent (M-H Index ranges from 0.991 (M2) to 0.9992 (M1) (Figure S4 Panel E). Same conclusions as closed-reference picking
- Analysis of the relative abundance of each of these organisms over time shows an increase in relative abundance for *Acinetobacter* (p = 0.004), *Mycobacterium* (p = 0.002), *Pseudomonas* (p = 0.015), *Sphingomonas* (p = 0.025), and *Stenotrophomonas* (p = 0.03) as treatment progressed (Figure S5). Whereas *Brevundimonas*, *Chryseobacterium*, and Legionellaceae did not demonstrate an increase in abundance following treatment (Figure S5). Same conclusions as closed-reference picking with the addition of the opportunistic pathogen containing genus *Stenotrophomonas* spp.
- There was no statistically significant difference in genera containing nitrifying bacteria, *Nitrospira* and Nitrosomonadaceae, before (mean = 0.0011 ± 0.0013) and after treatment (mean = 0.0005 ± 0.0011) (p = 0.388) (Figure S6). No other nitrifying bacteria of the genera *Nitrosococcus*, *Nitrobacter*, *Nitrospina*, or *Nitrococcus*, were found in any of our samples. The total relative abundance of the genera containing denitrifying bacteria *Thiobacillus, Micrococcus*, and *Paracoccus* underwent a statistically significant increase in the relative abundance of genera containing denitrifying bacteria before (mean = 0.00017 ± 0.00028) and after treatment with monochloramine (mean = 0.0025 ± 0.0022) (p = 0.0003) (Figure S6). Other denitrifying genera (*Rhizobiales* and *Rhodanobacter*) were not found in our samples. Same conclusions as closed-reference picking.

**Supplementary Reference:**

1. Duda S, Kandiah S, Stout JE, Baron JL, Yassin MH, et al. (2014) Evaluation of a new monochloramine generation system for controlling *Legionella* in building hot water systems. Submitted for publication.
